# Occurrence and localization of FOXP3 + cells in kidney biopsies in lupus nephritis and ANCA-associated vasculitis

**DOI:** 10.1007/s10067-023-06676-8

**Published:** 2023-06-27

**Authors:** Agneta Zickert, Marija Ratković Janković, Vivianne Malmström, Karine Chemin, Iva Gunnarsson

**Affiliations:** 1 Department of Medicine, Rheumatology Unit, Karolinska Institutet, Solna, Sweden; 2 Rheumatology Unit, S-171 76, Karolinska University Hospital, Stockholm, Sweden; 3 Department of Nephrology, Clinic of Pediatrics, University Clinical Center Nis, Medical Faculty, University of Nis, Nis, Serbia

**Keywords:** ANCA-associated vasculitis, FOXP3 + cells, Kidney biopsies, Lupus nephritis

## Abstract

The study aims to increase the understanding regarding the role of regulatory T cells (Tregs) in lupus nephritis (LN) and ANCA-associated vasculitis (AAV) by comparing their localization in renal tissue and changes following immunosuppressive therapy. Kidney biopsies from 12 patients with LN and 7 patients with AAV were examined. Kidney biopsies had been performed both at active disease and following immunosuppressive treatment. Clinical data was collected at both biopsy occasions. Expression of Forkhead Box P 3 (Foxp3) in renal tissue was assessed by immunohistochemistry. An arbitrary scale was used to estimate the number of Foxp3+ cells. In LN, 8/12 (67%) had positive tissue staining for Foxp3 at baseline, most pronounced in inflammatory infiltrates, but also interstitially and in a peri-glomerular pattern. At second biopsies, after immunosuppressive treatment, 4/12 (33%) still had detectable Foxp3+ cells, found in persisting inflammatory infiltrates and some in the interstitium. Patients with a good clinical response to treatment had high grade of Foxp3+ cells in first biopsies. In AAV, only 2/7 (29%) had positive staining for Foxp3 at baseline, in inflammatory infiltrates and to a lesser extent in the interstitium, despite large areas of inflammatory infiltrates in all patients. At follow-up, 2/7 (29%) biopsies were positive for Foxp3. Our data show a higher presence of Foxp3+ cells in renal tissue from LN patients compared to AAV, suggesting that Tregs may be differently involved in the control of inflammatory mechanisms in these diseases. These findings could have further implication for therapeutic approaches aiming at restoring the immunological tolerance.

**Table:** 

**Key Points** *• Foxp3+-cells are present in larger amount in renal tissue in lupus nephritis vs. ANCA-associated vasculitis.* *• Our data suggest that Foxp3+ regulatory T cells are involved in the control of inflammatory processes in lupus nephritis.*

**Supplementary Information:**

The online version contains supplementary material available at 10.1007/s10067-023-06676-8.

## Introduction

Lupus nephritis (LN) is a severe manifestation of systemic lupus erythematosus (SLE), affecting up to 60% of the patients at some point of the disease [1]. The pathogenesis for LN is not completely understood and involves multiple components of the immune system. One hallmark in the pathogenesis for SLE is loss of self-tolerance [2]. Specific subsets of CD4^+^ cells, the regulatory T cells (Tregs), have regulatory and immunosuppressive functions, mainly by suppressing self-reactive T cells and are central for maintaining peripheral tolerance. Tregs have been proposed to be involved in the pathogenesis for various autoimmune diseases including SLE [3]. In SLE, an impairment of both the function and the number of Tregs have been documented and are believed to be central in the pathogenesis [2]. Forkhead Box P3 (Foxp3) is a transcription factor that is necessary for the development and function of Tregs [4]. The expression of Foxp3 is generally used as a marker to identify and study Tregs [3].

Anti-neutrophil cytoplasmic antibody (ANCA)-associated vasculitis (AAV) is a group of vasculitides commonly affecting the kidneys [5, 6]. Renal involvement in AAV gives rise to a necrotizing glomerulonephritis which, if untreated, leads to a severe inflammatory state with rapid deterioration of kidney function [6]. Several immune cells are involved in the inflammatory process in AAV. Neutrophils play a central role in the pathogenesis and both B cells and T cells are activated and expanded, while the immunosuppressive function of Tregs have been shown to be impaired [7].

However, there is limited data on the presence of Tregs in the target organs in both SLE and AAV. It is not known whether the amounts of Tregs in renal tissue are more pronounced in active renal disease, or how they are affected by immunosuppressive treatment in these diseases.

In this study, we performed immunohistochemistry staining of Foxp3 in renal tissue from LN and AAV patients, both at an active disease state and after immunosuppressive treatment, and studied their precise distribution and any correlation with the extent of inflammatory cell infiltrates. Thus, we aimed to increase the understanding on the role of Tregs in inflammatory autoimmune kidney diseases.

## Methods

### Patients

The study consisted of 38 kidney biopsies from 19 patients with SLE or AAV. Twelve patients with LN confirmed by a kidney biopsy, and in whom second biopsies were performed after induction immunosuppressive treatment, were included in the study. All patients met the 1982 American College of Rheumatology (ACR) classification criteria for SLE [8], and the SLICC criteria from 2012 [9]. After the initial kidney biopsy performed in active disease state, the patients were treated with either cyclophosphamide (CYC) (*n* = 6) or mycophenolate mofetil (MMF) (*n* = 6) (Table 1), and all patients were also treated with prednisolone, median dose 35 mg daily (range 10–60). Repeated biopsies were performed after a median time of 7 months (range 6–13).

**Table 1:** Clinical, laboratory, and histopathological characteristics in lupus nephritis patients at first and second biopsies

	First biopsy	Second biopsy	*p*-Value
Gender, *n* (%)			
Female	10 (83%)		
Male	2 (17%)		
Age	36 (21–50)		
Creatinine (μmol/l)	70 (40–96)	67 (39–98)	ns
Albuminuria (g/day)	1.2 (0.01–5.6)	0.1 (0.01–0.9)	0.008
Anti-DNA ab pos	7/12	5/12	ns
C3 (g/l)	0.4 (0.3–1.13)	0.7 (0.36–1.41)	ns
C4 (g/l)	0.06 (0.02–0.39)	0.13 (0.02–0.45)	0.02
Renal histology (ISN/RPS), *n*			
Class II	-	3	
Class III-A or A/C	4	1	
Class IV-A or A/C	3	-	
Class III/IV + V	3	-	
Class V	2	7	
Vasculitis	-	1	
Prednisolone at biopsy dose (mg/day)	12.5 (0–30)	10 (5–20)	
Induction treatment, *n*			
Cyclophosphamide	6		
Mycofenolate mofetil	6^*^		

Values are presented as median (range) unless otherwise indicated

*n* = number of patients, *C3 and C4* = complement components 3 and 4, *ISN/RPS* = International Society of Nephrology/Renal Pathology Society classification

^*^One patient in the mycophenolate (MMF) group had initially received 2 doses of cyclophosphamide (CYC) and was switched to MMF because of an infusion reaction to CYC

As comparator, seven patients with AAV from the Karolinska Vasculitis cohort (VASKA) who had a kidney biopsy performed at active phase of disease and then undergone a second biopsy after immunosuppressive treatment were included. All biopsies were performed as protocol biopsies. The median time between first and repeated biopsy was 7 (range 5.5–9) months. Four patients had a diagnosis of granulomatosis with polyangiitis (GPA) and 3 had microscopic polyangiitis (MPA). After the initial biopsy, the patients were treated with cyclophosphamide (CYC) (*n* = 6) or azathioprine (AZA) (*n* = 1) (Table 2) and all patients were also treated with prednisolone.

**Table 2:** Clinical, laboratory and histopathological characteristics in ANCA-associated vasculitis patients at first and second biopsies

	First biopsy	Second biopsy	*p*-Value
Gender, *n* (%)			
Female	6 (86%)		
Male	1 (14%)		
Age (range)	58 (44–79)		
Creatinine, μmol/l (range)	100 (73–900)	98 (73–119)	ns
Urine findings, positive *n* (%)			
Albuminuria (dip slide)	6 (100%)^*^	2 (33%)^*^	ns
Hemoglobinuria (dip slide)	6 (100%)^*^	2 (33%)^*^	0.04
Antibody positivity, *n*			
PR-3^**^	4		
MPO^***^	3		
Renal histology (Berden), *n*			
Crescentic	2	-	
Focal	5	-	
Prednisolone at biopsy dose, mg/day (range)	40 (0–60)	10 (7.5–15)	
Induction treatment, *n*			
Cyclophosphamide	5		
Methotrexate	1		
Azathioprine	1		

Values are presented as median (range) unless otherwise indicated

*n* = number of patients

^*^Missing data from one patient

^**^Antibodies against proteinase 3

^***^Antibodies against myeloperoxidase

In both LN and AAV patients, clinical data including blood and urinary findings had been collected at both biopsy occasions. Written informed consent was obtained from all subjects and the regional ethics committee in Stockholm approved the study protocol.

### Evaluation of kidney function and renal disease activity

Renal evaluation included urine analyses (dip slide procedure), determination of plasma creatinine (µmol/l), and in LN patients also investigation of 24-h urine-albumin excretion or u-albumin/creatinine ratio (mg/mmol). A good clinical renal response to treatment in LN patients was defined as having albuminuria < 0.5 g/day at second biopsy [10].

### Serology and complement measures

Anti-dsDNA antibodies were analyzed by immunofluorescence microscopy using *Crithidiae luciliae* as source of antigen. The complement components C3 and C4 were determined by nephelometry. Analyses of PR3-and MPO antibodies were performed according to clinical routine at the Department of Clinical Immunology at the Karolinska University Hospital.

### Histopathological evaluation

The kidney biopsies were evaluated by light microscopy, immunofluorescence, and electron microscopy. Biopsies from the LN patients were classified according to the International Society of Nephrology/Renal Pathology Society (ISN/RPS) classification [11]. The biopsies were also scored for activity and chronicity indices, an established method for evaluation of different signs associated with active inflammation and damage in renal tissue [12].

In AAV, the renal involvement was classified in different subtypes according to Berden et al. [13]. A histopathological response to treatment in proliferative LN was defined as > 50% reduction of activity index at second biopsy. In membranous LN (ISN/RPS class V), we defined histological response as an increased resorption of immune deposits on electron micrographs [14].

### Staining procedure

Immunohistochemical staining of Foxp3 was performed on formaldehyde-fixed paraffin-embedded sections of renal biopsies, using a murine recombinant anti-FOXP3 antibody clone (Abcam, ab20034) in a Ventana automated immunohistochemistry system (Ventana Medical Systems, Tucson, AZ) at the Department of Clinical Pathology. For CD3 staining, anti-CD3 antibodies were used (Thermo Scientific, Rockford, IL, USA).

An arbitrary scale for evaluation of the amount of Foxp3 + cells was used, ranging from 0 to 2 (0 = no, 1 = some, or 2 = plenty). The biopsies were evaluated by two investigators and were then scored in consensus. The evaluation was done blinded regarding diagnosis, clinical data, and if the slides were from first or second biopsies.

### Statistics

We performed Wilcoxon matched pair test to compare variables at baseline and follow-up and Mann–Whitney test for comparisons between two groups. Correlations were calculated using Spearman’s rank correlation. Statistical significance was set at the level of *p* < 0.05. Statistical evaluation was performed by statistical software, STATISTICA, StatSoft, USA.

## Results

### Histopathology and renal activity

In LN, all patients had an active nephritis at baseline, class III-A or A/C (*n* = 4), class IV-A or A/C (*n* = 3), class III–IV/V (*n* = 3), or class V (*n* = 2). Follow-up biopsies revealed class II (*n* = 3), class III A/C (*n* = 1), or class V (*n* = 7). One patient developed a renal vasculitis.

Creatinine levels remained stable at second biopsy, while a decrease in albuminuria was seen (*p* = 0.008) (Table 1).

According to the definition used, 9/12 (75%) of the patients had a good clinical response to treatment, and 8/12 (67%) were regarded histopathological responders.

At baseline, all AAV patients had an active renal vasculitis. According to the classification system used [13], two patients had crescentic and five had focal vasculitis. Follow-up biopsies were all improved with no signs of active vasculitis; however, an increase in chronic changes was seen. AAV patients had higher creatinine levels than LN patients at both first and second biopsies (*p* = 0.016 and 0.005, respectively). However, no overall difference in creatinine levels between baseline and follow-up was found in the AAV group (Table 2).

### Foxp3 expression in renal tissue in LN and AAV

**Fig. 1:**
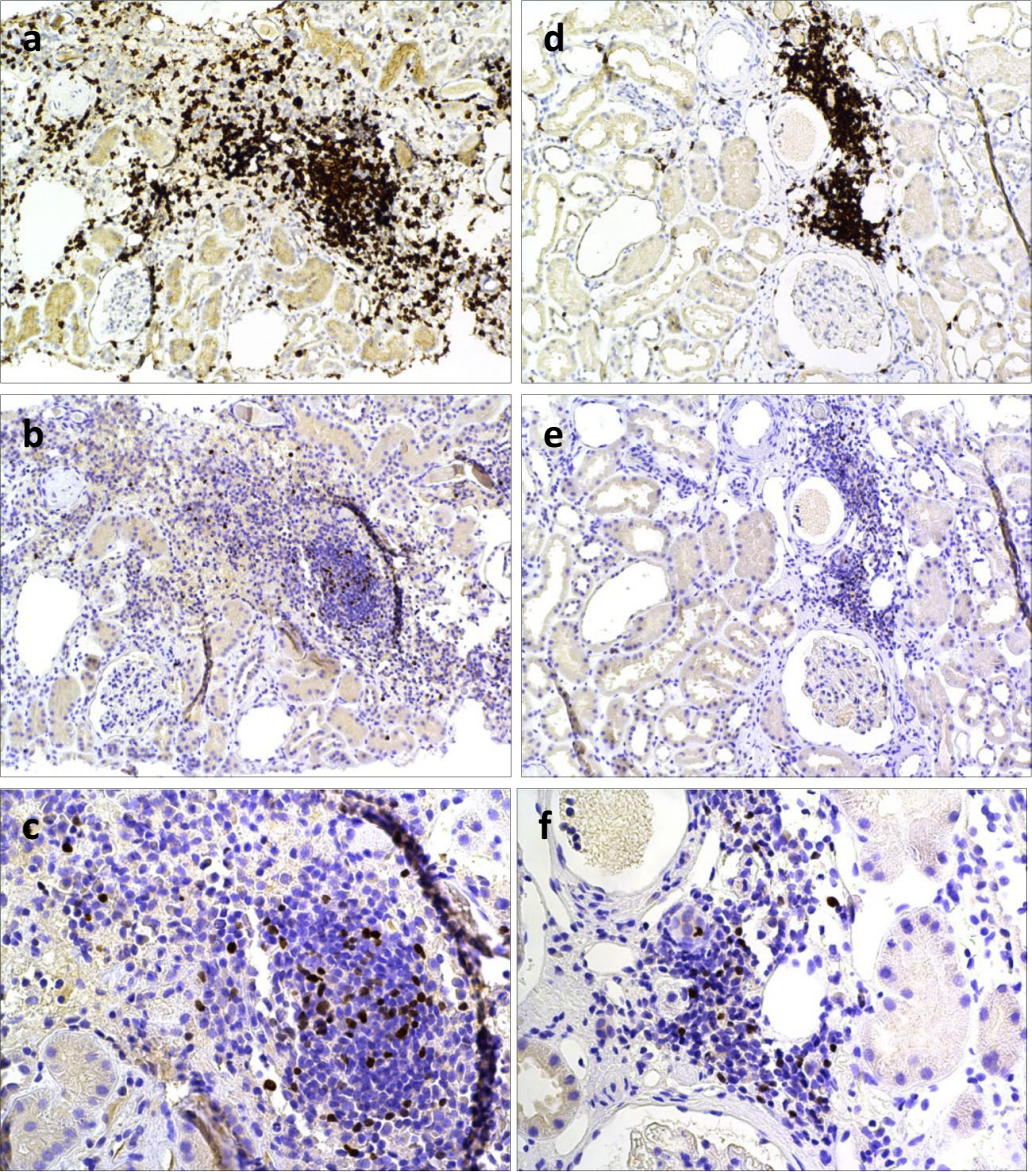
Immunostaining of Foxp3 in renal tissue in lupus nephritis. The figure demonstrates kidney biopsies at active disease (**a–c**) and after immunosuppressive treatment (**d–f**) from a patient with lupus nephritis ISN/RPS class III (A). Representative micrographs displaying (**a**) an inflammatory infiltrate with T cells demonstrated by positive CD3 staining and (**b** and **c**) positive staining for Foxp3 predominantly found in the same infiltrate. A repeat biopsy (ISN/RPS class II) from the same patient reveals a persisting inflammatory infiltrate with CD3 + cells (**d**) and positive Foxp3 stainings (**e** and **f**) in the same infiltrate, although to a lesser extent. Original magnifications: 10 × (**a**, **b**, **d**, **e**) and 25 × (**c**, **f**)

At first biopsy, 8/12 (67%) of LN patients had positive tissue staining for Foxp3. The expression of Foxp3 was most pronounced in CD3 + inflammatory infiltrates, but also found in the interstitium and in a periglomerular pattern. At second biopsies, after immunosuppressive treatment, 4/12 (33%) had detectable Foxp3 + cells, all found in persisting inflammatory infiltrates and to a lesser extent also interstitially (Fig. 1). Overall, 5 patients had decreased, 2 had unchanged, and 3 had increased amounts of Foxp3 positive cells, as defined by the scoring system used.

**Fig. 2:**
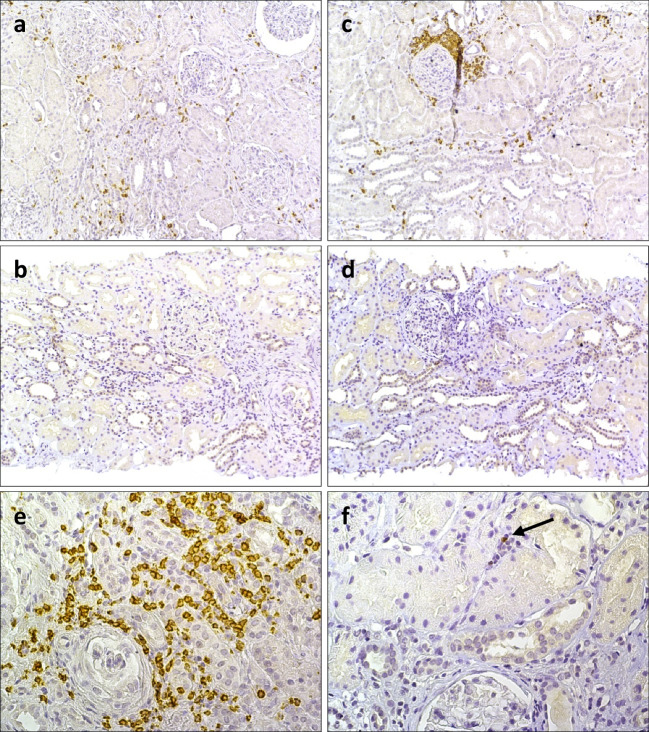
Immunostaining of Foxp3 in renal tissue in AAV. The figure demonstrates kidney biopsies at active disease (**a** and **b**) and after immunosuppressive treatment (**c–f**) from a patient with microscopic polyangiitis with renal vasculitis. Representative micrographs displaying (**a**) an inflammatory infiltrate with T cells demonstrated by positive CD3 staining and in (**b**) absence of Foxp3 + cells. A repeat biopsy from the same patient reveals a periglomerular inflammatory infiltrate with CD3 + cells (**c**), although a negative Foxp3 staining (**d**). Higher magnification of a micrograph of renal tissue from the same repeat biopsy shows an inflammatory infiltrate (**e**), with a single cell positive for Foxp3 staining (**f**). Original magnifications: 10 × (**a**, **b**, **c**, **d**) and 25 × (**e**, **f**)

In AAV patients, 2/7 (29%) had positive tissue staining for Foxp3 at baseline, most pronounced in inflammatory infiltrates and to a lesser extent in the interstitium. All patients had areas of inflammatory infiltrates of CD3 + T cells. At follow-up, 2/7 patients were still positive for Foxp3 (Fig. 2). A detailed description of findings from the tissue staining is presented in supplementary table 1.

### Association between Foxp3 expression and clinical findings in LN patients

Patients with a good clinical treatment response had higher numbers of Foxp3 + cells in the renal tissue at first biopsies (*p* = 0.02) compared to non-responders (Supplementary table 2). In patients with a good clinical response, 8/9 (89%) had a positive Foxp3 staining at first biopsies whereas none of the clinical non-responders had a positive Foxp3 staining. Furthermore, levels of albuminuria at second biopsies correlated negatively to the initial number of Foxp3 + cells (*r* =  − 0.64, *p* < 0.05) while there was a positive correlation between baseline Foxp3 + to C4 at follow up (*r* = 0.7, *p* < 0.05).

Patients with a histopathological response to treatment had a trend of higher numbers of Foxp3 + cells at first biopsies (*p* = 0.052). We found no clear association between the grade of Foxp3 expression in renal tissue and creatinine levels, type of LN, or to immunosuppressive treatment given (data not shown).

## Discussion

We found Foxp3-expressing cells in renal tissue from a majority of the LN patients, most pronounced in inflammatory infiltrates with CD3 + cells, but also as solitary cells in the interstitium and in periglomerular areas. In AAV patients, although having large areas of CD3 + infiltrates, only a few had Foxp3-positive cells both at baseline and in follow-up biopsies. Thus, we here confirm a disease-specific presence of Foxp3-expressing cells at a tissue level in LN supporting the hypothesis that regulatory T cells are involved in the inflammatory process.

We found Foxp3 expression in most of the biopsies from LN patients, especially at active disease. We also observed a decrease following therapy. Interestingly, patients with a good clinical treatment response had significantly higher grade of Foxp3 + cells, whereas all patients with a poor clinical response had a negative Foxp3 + staining, suggesting a role for Tregs in controlling the inflammatory response. There was also a trend that a favorable histopathological response was seen in patients with a high grade of Foxp3 + cells. Although not significant, possibly due to the low sample size, this finding also supports that Tregs are involved in control of the inflammatory process in LN. In a previous study, increased expression of Foxp3 was found in kidney tissue from 50 patients with LN, where the grade of expression correlated with the severity of the disease [15]; however, in that study, there were no repeat kidney biopsies available. Increased levels of Foxp3 mRNA have also been reported in urine from patients with active LN compared to patients with inactive lupus and healthy controls. Interestingly, the Foxp3 mRNA levels also correlated with the activity index in kidney biopsies and was proposed as a biomarker for active renal disease [16].

Although several studies have documented low numbers or impaired function of circulating Tregs in lupus patients, other studies have demonstrated no difference or even higher levels of Tregs in SLE compared to controls [17]. These conflicting data could be due to many reasons including disease stage, disease severity, and treatment. Our observation that Foxp3 + cells are clearly identifiable in the LN kidney biopsies support the hypothesis that although suppressive Tregs exist at the site of inflammation, they may fail to control disease progression [18].

Treatment strategies aiming to restore the immune tolerance in SLE by manipulation of Tregs have been proposed in many recent studies. In SLE the production of interleukin-2 (IL-2), a cytokine that is important for the differentiation and function of Treg, is disturbed [2]. Clinical trials with low-dose IL-2 in lupus, given to expand, restore, and improve the function of Tregs, have shown promising results [2, 19, 20].

The limitations of the study are the limited number of patients and that is a retrospective and observational study with different intervals between biopsies, as decided by the treating physician, and where treatment strategies have changed over time. However, the study was performed in a clinical setting and thus reflects real-life care which may also be a strength.

Although this is a small study which impedes any firm conclusions, our findings suggest that Tregs are involved in the control of inflammatory processes in LN but seem to be of less importance in AAV. Different mechanisms in Treg generation or migration at inflammatory sites in these two diseases might explain these differences but further investigation will be needed in larger cohorts. The relative absence of FOXP3 + T cells in AAV is surprising given the notion that Tregs tend to accumulate in inflammatory infiltrates in various tissues and diseases. The underlying reasons for this warrant further studies of AAV.

Overall, our findings support further studies of therapeutic approaches aiming at restoring the immunological tolerance in SLE.

### Supplementary Information

Below is the link to the electronic supplementary material.

Supplementary file1 (DOCX 20 KB)
