# Supplementary material for: Occurrence and localization of FOXP3 + cells in kidney biopsies in lupus nephritis and ANCA-associated vasculitis
Source: Clin Rheumatol. 2023 Jun 27;42(10):2889–95. doi: 10.1007/s10067-023-06676-8 (PMC10497686; doi:10.1007/s10067-023-06676-8)
Supplement: Supplementary file 1 — Supplementary file1 (DOCX 20 KB) [file 10067_2023_6676_MOESM1_ESM.docx]

**Supplementary table 1. Foxp3 immunohistochemistry stainings in first and second kidney biopsies**

| LN/  AAV | Foxp3, biopsy 1 | grade | Foxp3, biopsy 2 | grade | Localisation Foxp3,  biopsy 1 | Localisation Foxp3,  biopsy 2 |
| --- | --- | --- | --- | --- | --- | --- |
| LN | pos | 1 | neg | 0 | infiltrate, interstitium, periglomerular | - |
| LN | neg | 0 | neg | 0 | - | - |
| LN | pos | 1 | neg | 0 | infiltrate, periglomerular | - |
| LN | neg | 0 | neg | 0 | - | - |
| LN | neg | 0 | neg | 0 | - | - |
| LN | neg | 0 | pos | 1 | - | infiltrate |
| LN | pos | 1 | neg | 0 | capillaries | - |
| LN | pos | 1 | pos | 2 | interstitium, periglomerular | infiltrate, interstitium |
| LN | pos | 1 | pos | 2 | infiltrate, interstitium | infiltrate |
| LN | pos | 2 | pos | 2 | infiltrate, interstitium | infiltrate, interstitium |
| LN | pos | 1 | neg | 0 | interstitium, capillaries | - |
| LN | pos | 2 | neg | 0 | infiltrate, interstitium, periglomerular | - |
| GPA | neg | 0 | neg | 0 | - | - |
| GPA | neg | 0 | pos | 1 | - | infiltrate |
| MPA | pos | 1 | pos | 1 | interstitium | interstitium |
| MPA | neg | 0 | neg | 0 | - | - |
| GPA | neg | 0 | neg | 0 | - | - |
| GPA | pos | 2 | neg | 0 | infiltrate | - |
| MPA | neg | 0 | neg | 0 | - | - |

Results from Foxp3 immunohistochemistry staining of renal tissue from lupus nephritis (LN) and ANCA-associated vasculitis (AAV) patients at active disease and after immunosuppressive therapy, GPA= Granulomatosis with polyangiitis, MPA= Microscopic polyangiitis. The grade of staining is demonstrated according to an arbitrary scale (0= no; 1= some or 2= plenty).

**Supplementary table 2. Foxp3 immunohistochemistry staining from first and second biopsies in lupus nephritis (LN) patients.**

| \| Diagnos \| \| --- \| | \| Foxp3  biopsy 1 \| \| --- \| | \| grade \| \| --- \| | \| Foxp3  biopsy 2 \| \| --- \| | \| grade \| \| --- \| | \| ISN/RPS \| \| --- \|   biopsy 1 | \| ISN/RPS \| \| --- \|   biopsy 2 | \| HR \| \| --- \| | \| CR \| \| --- \| |
| --- | --- | --- | --- | --- | --- | --- | --- | --- | --- | --- | --- | --- | --- | --- | --- | --- | --- |
| LN | pos | 1 | neg | 0 | III (A/C) | II | yes | yes |
| LN | neg | 0 | neg | 0 | V | V | no | no |
| LN | pos | 1 | neg | 0 | III (A)/V | V | yes | yes |
| LN | neg | 0 | neg | 0 | III (A) | III A/C | no | yes |
| LN | neg | 0 | neg | 0 | III (A) | II | yes | no |
| LN | neg | 0 | pos | 1 | V | V | no | no |
| LN | pos | 1 | neg | 0 | IV G (A) | V | yes | yes |
| LN | pos | 1 | pos | 2 | IV S (A/C) | vaskulit | no | yes |
| LN | pos | 1 | pos | 2 | III/V | V | yes | yes |
| LN | pos | 2 | pos | 2 | III (A) | II | yes | yes |
| LN | pos | 1 | neg | 0 | IV G (A) +V | V | yes | yes |
| LN | pos | 2 | neg | 0 | IV G (A) | II/V | yes | yes |

Results from Foxp3 immunohistochemistry staining of renal tissue from LN patients at active disease and after immunosuppressive therapy, the grade of staining is demonstrated according to an arbitrary scale (0= no; 1= some or 2= plenty). ISN/RPS; classification of type of LN, HR; histopathological response to treatment, CR; clinical response to treatment
